# The Concept of Cancer Stem Cells: Elaborating on ALDH1B1 as an Emerging Marker of Cancer Progression

**DOI:** 10.3390/life13010197

**Published:** 2023-01-09

**Authors:** Ilias Tsochantaridis, Angelos Roupas, Sofie Mohlin, Aglaia Pappa, Georgia-Persephoni Voulgaridou

**Affiliations:** 1 Department of Molecular Biology & Genetics, Democritus University of Thrace, 68100 Alexandroupolis, Greece; 2 Division of Pediatrics, Clinical Sciences, Lund Stem Cell Center, Lund University Cancer Center, 22384 Lund, Sweden

**Keywords:** cancer, aldehyde dehydrogenases, ALDHs, aldehyde dehydrogenase 1B1, cancer stem cells, CSCs

## Abstract

Cancer is a multifactorial, complex disease exhibiting extraordinary phenotypic plasticity and diversity. One of the greatest challenges in cancer treatment is intratumoral heterogeneity, which obstructs the efficient eradication of the tumor. Tumor heterogeneity is often associated with the presence of cancer stem cells (CSCs), a cancer cell sub-population possessing a panel of stem-like properties, such as a self-renewal ability and multipotency potential. CSCs are associated with enhanced chemoresistance due to the enhanced efflux of chemotherapeutic agents and the existence of powerful antioxidant and DNA damage repair mechanisms. The distinctive characteristics of CSCs make them ideal targets for clinical therapeutic approaches, and the identification of efficient and specific CSCs biomarkers is of utmost importance. Aldehyde dehydrogenases (ALDHs) comprise a wide superfamily of metabolic enzymes that, over the last years, have gained increasing attention due to their association with stem-related features in a wide panel of hematopoietic malignancies and solid cancers. Aldehyde dehydrogenase 1B1 (ALDH1B1) is an isoform that has been characterized as a marker of colon cancer progression, while various studies suggest its importance in additional malignancies. Here, we review the basic concepts related to CSCs and discuss the potential role of ALDH1B1 in cancer development and its contribution to the CSC phenotype.

## 1. Introduction

Cancer is a term used to describe a wide panel of diseases that mainly involve uncontrolled cellular proliferation, and in aggressive cases, the metastatic invasion of cancer cells to the circulatory system and eventually to new, healthy tissue(s) [1]. It is a major global health issue, considering that in 2021, 1,898,160 new cases of cancer as well as 608,570 cancer-related deaths occurred in the United States of America alone [2]. Several risk factors are suggested to be associated with the development of cancer, including inflammation, aging, smoking, growth factors, hormones, radiation, alcohol, diet, and obesity [3].

**Figure 1:**
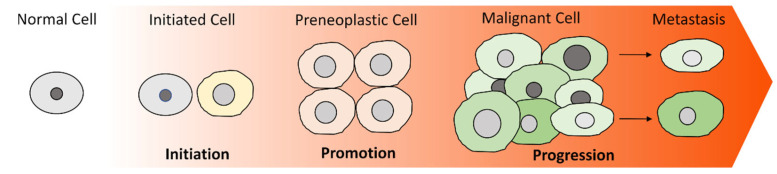
The process of malignant transformation. Cancer is a multi-staged procedure in which cells gradually acquire malignant characteristics. Initiation includes certain genetic/epigenetic changes resulting in the deregulated control of processes, such as cell-cycle progression, apoptosis, and proliferation. The clonal expansion of the initiated cell, which exhibits defective apoptosis, abnormal cell-cycle arrest, and excessive proliferation, leads to the formation of a preneoplastic lesion of closely attached cells. During progression, the genetically unstable preneoplastic cells progressively accumulate novel, malignant-related properties, such as the ability to escape from immune surveillance, migrate and invade new tissues, and form new tumors.

Cancer is defined by 14 different hallmarks, including its limitless replicative potential, constant proliferative signaling, ability to evade cell death and growth suppressors, activation of angiogenesis or access to the vasculature, induction of tissue invasion and metastasis, reprogramming of metabolism, escape from immune destruction, tumor-inducing inflammation, and genetic instability. Additionally, properties that are related to and enable cancer growth are the abilities of phenotypic plasticity, cellular senescence, polymorphism of microbiomes, and epigenetic alterations [1,4,5]. The process by which a non-malignant cell gradually acquires cancer-promoting characteristics is termed malignant transformation or carcinogenesis. Carcinogenesis is a long, complex and multi-step procedure that can be categorized, in a simplified, conceptual manner, into three main stages: initiation, promotion, and progression (Figure 1) [6].

Initiation is an irreversible process that involves the deregulation of one or more genes associated with crucial regulatory pathways, either by chromosomal genetic or epigenetic alterations. These genes are considered to be either oncogenes, the activation of which results in increased cellular proliferation, or tumor-suppressing genes, the inhibition of which induces the inactivation of cell-cycle arrest and/or apoptosis [7,8]. A malfunction of these genes leads to the transformation of a healthy cell into a preneoplastic cell [9,10]. An important aspect of cancer research is identifying the origins of cancer, i.e., the first cell(s) acquiring preneoplastic properties and the molecular context that supports and drives this biological process. Up until now, the origins of many cancers remain elusive. Recent experimental data report that while both stem and non-stem cells can initiate malignant transformation, the capacity to do so depends on the type of tissue and resulting cancer [11,12,13,14,15].

Promotion refers to the selective clonal growth of the preneoplastic, initiated cell and its progeny as a result of their ability to evade apoptosis and/or their enhanced cell proliferation. At the promotion phase, the expanded clone of the preneoplastic cells forms a benign tumor in which the cells remain in close contact with each other; thus, they cannot detach from one another [9,16].

Finally, during progression, the preneoplastic cells acquire a neoplastic phenotype by progressively acquiring additional malignant-related characteristics, such as invasiveness and metastatic potential. It has been proposed that the acquisition of these properties could be attributed to various factors, such as genetic/chromosomal instability and epigenetic deregulation. At this stage, certain cancer cells have acquired the capacity to detach from their initial location, invade nearby or distant tissues and, consequently, lead to the formation of secondary tumors (metastasis) [16,17]. Interestingly, the now malignant tumor comprises cancer cells with various genetic, epigenetic, morphological, and metabolic properties that exhibit significant differences in their proliferation, tumorigenic, and metastatic potential. Thus, tumors comprise distinct and highly heterogeneous cell populations [18].

The phenotypic and genomic diversity of cancer constitutes, perhaps, one of the greatest challenges to overcome for improving current clinical approaches as well as for developing novel, efficient therapeutic strategies. To this day, most anticancer protocols manage to kill bulk tumor populations efficiently, however, they fail to target all the different cancer cell types [19]. Considering the detrimental clinical effects of intratumoral heterogeneity (e.g., chemo/radio-resistance, increased aggressiveness, metastasis, and disease recurrence), it is of crucial importance to investigate the underlying mechanism(s) [20].

## 2. Cancer Stem Cells (CSCs)

**Figure 2:**
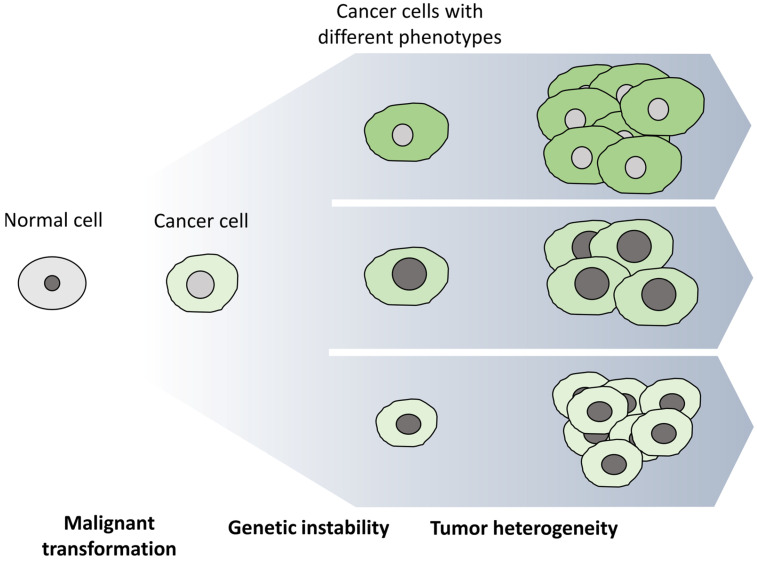
The clonal or stochastic cancer development model. In the clonal model, every cancer cell has the potential to promote the development of a new tumor. The driving force of tumor heterogeneity is the genetic instability of cancer cells that results in the accumulation of DNA alterations and, consequently, the formation of cancer cells with different genotypes and, thus, phenotypes.

**Figure 3:**
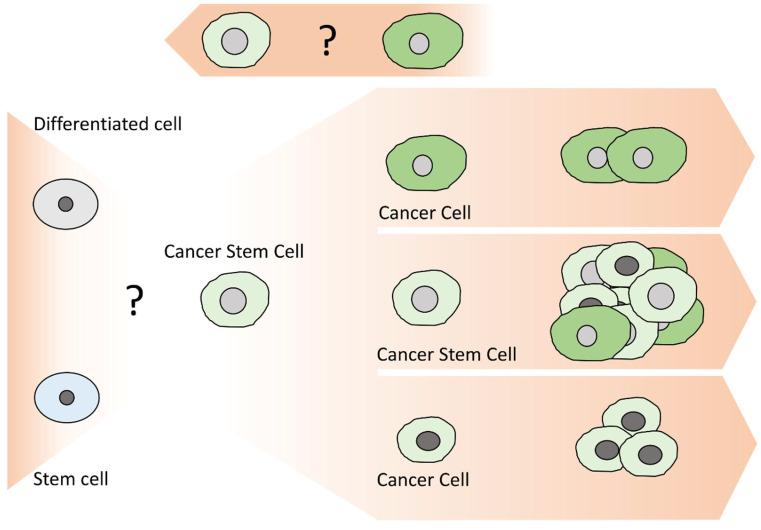
The hierarchical or cancer stem cell development model. In the hierarchical model, cancer stem cells are able to self-renew and differentiate into multiple, different cancer cell types and, thus, induce the formation of a new tumor with the same heterogeneity as the initial one. Some studies support the existence of distinct CSC subpopulations within a tumor. An important aspect that has not yet been clarified is whether CSCs originate from normal stem cells undergoing malignant transformation or whether they derive from differentiated cells that acquire stem-related properties during carcinogenesis. Finally, certain reports demonstrate the plasticity of CSCs, which appear to have the potential to switch between the CSC and the non-CSC states through unknown mechanisms.

The diversity of cancer cells in the same tumor bulk has led to the development of several theories for explaining tumor progression. Among them, there are two prevailing models that do not necessarily contradict one another: the clonal or stochastic and the hierarchical or cancer stem cell models [21,22,23,24]. According to the clonal model (Figure 2), the genetic and epigenetic instability of cancer cells is the driving force of tumor heterogeneity [25]. Consequently, all cancer cells initially have the same capacity to acquire the properties required for leading the formation of a new tumor [26]. On the other hand, the CSC model (Figure 3) designates that tumors are organized in a hierarchical manner, similar to that of a normal tissue, in which only a small subset of cells, CSCs, are responsible for driving tumor development as well as leading to metastasis and/or disease recurrence [27,28].

Cancer stem cells attribute their name to the stem-like properties they possess, such as their self-renewal potential, asymmetrical cell division, multi-lineage differentiation capacity, ability to remain in a non-differentiated-quiescent state, potential to form new heterogeneous tumors, and their capability to exhibit phenotypic plasticity [29,30,31]. CSCs were first identified in an immunodeficient NOD/SCID mouse model of acute myeloid leukemia (AML) [32]. Specifically, a CD34+/CD38- sub-population, which constituted approximately 0.1–1% of the total population of leukemic cells, was found to exhibit properties similar to that of normal stem cells in addition to having the ability to initiate AML [32]. Since then, CSCs and their tumorigenic potential have been identified in a wide spectrum of both hematopoietic and solid malignancies [33].

CSCs have been the subject of extensive studies over the past several years; nevertheless, their origin remains unclarified, and it is unknown whether they originate from normal stem cells undergoing neoplastic transformation or whether their stem-like properties derive from the accumulation of genetic alterations in differentiated cancer cells [34,35]. The concept of CSCs becomes even more complex when considering that, within the same tumor, various subpopulations of CSCs with distinct phenotypes and properties are able to co-exist. Furthermore, newly presented data describe CSCs as a dynamic rather than a static population, having the ability to inter-convert both between the CSC and non-CSCS states, as well as between the different CSC phenotypes through unclarified mechanisms [36,37,38,39,40].

Even though CSCs represent only a small proportion of cancer cells, they have an important impact on the clinical course of the disease due to their increased radio/chemo- resistance as well as their enhanced tumorigenic and metastatic abilities. Conventional therapeutic approaches do not effectively target the CSC population, and these cells can then initiate new tumors and, consequently, lead to disease recurrence and/or metastasis [41,42].

### 2.1. Methods for Isolating CSCs

**Figure 4:**
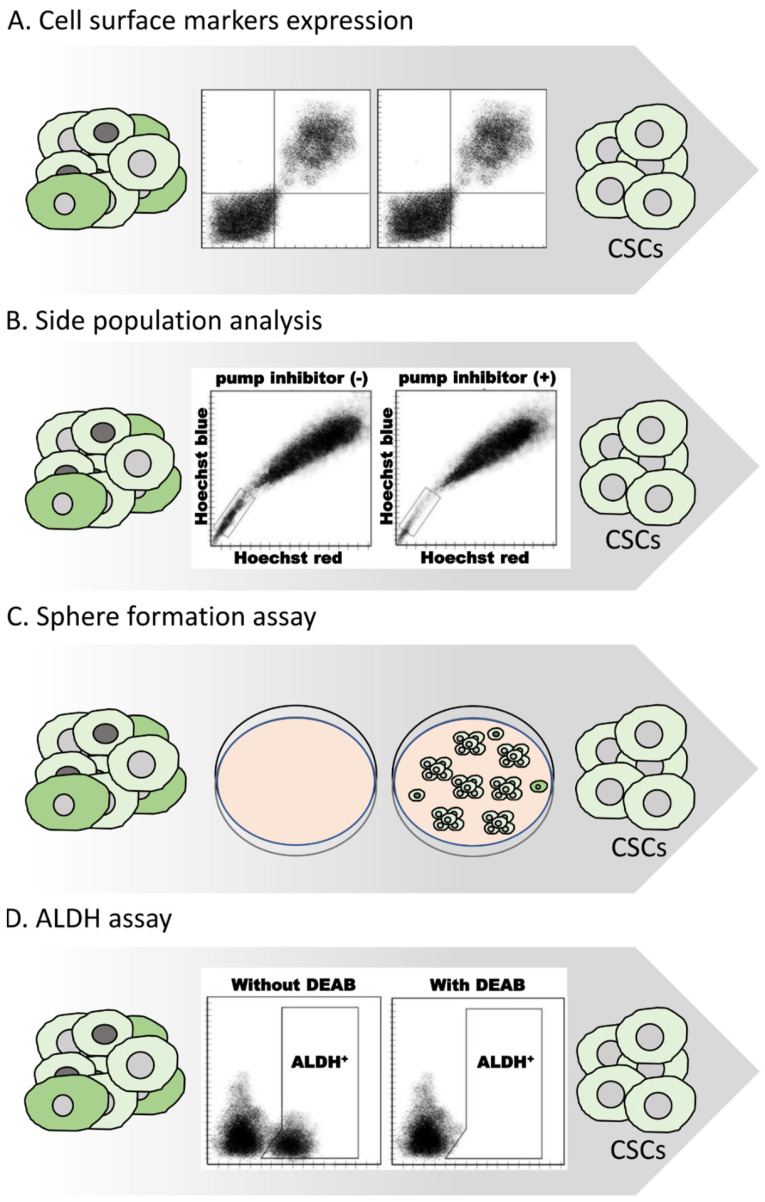
Methods for isolating CSC-like cells. A variety of different methods have been developed for isolating and enriching CSCs from different samples. Often these methods are combined to achieve efficient isolation of CSCs. (**A**) Selection based on the expression of certain cell-surface markers. (**B**) Isolation based on the ability of cells to efflux certain stains. (**C**) Enrichment based on the formation of 3D tumorspheres. (**D**) Isolation through determining ALDH enzymatic activity.

Due to their high clinical importance, several methods have been developed for efficiently distinguishing and isolating CSCs from the tumor bulk. The most prevailing methods for CSCs’ isolation include the sorting of cells based on their cell-surface markers, a side-population (SP) analysis through Hoechst 33342 exclusion, a sphere formation assay and the utilization of ALDH enzymatic activity (Figure 4) [43,44].

A wide variety of specific transmembrane proteins, such as SSEA3, CD24, CD26, CD34, CD44, CD55, CD133 (or prominin-1), CD166, CD326 (or EpCAM), ABCG2, CD49f, and CXCR4 can be used as biomarkers for the isolation of CSC populations via fluorescence-activated cell sorting (FACS) as well as through the utilization of magnetic beads with immobilized antibodies (magnetic-activated cell sorting (MACS)) (Figure 4A) [33,45,46]. However, the expression of these markers is not universal. On the contrary, the expression of most of these markers is highly dependent on various factors, such as the type of cancer, the histotype of the tumor, and the cell model used (primary cell cultures, tumor specimens, or established cell lines). Additionally, certain technical aspects, for instance, the protocol applied for isolating and/or disassociating cancer cells or even the culturing conditions, are associated with the proteolysis of cell-surface proteins and, thus, affect the use of these markers. The FACS procedure itself can also induce stress and, consequently, abnormal cellular behavior [47,48,49,50,51]. Finally, the usage of different sets of cell-surface markers often leads to the isolation of CSCs with different characteristics. Due to these limitations, the use of cell-surface markers is most often used in combination with other isolation protocol methods [52,53,54].

Side-population (SP) analysis is a widely used assay for isolating cancer cells with the ability to efflux DNA-binding fluorescent dyes via the ATP-binding cassette (ABC) transporters, such as BCRP/ABCG2 and MDR1/ABCB1 (Figure 4B) [55,56,57]. Specifically, cancer cells are stained with Hoechst 33342 or Rhodamine 123 and then analyzed through flow cytometry. SP cells effectively pump out the dye and, thus, appear unstained. The analysis is repeated in the presence of a pump inhibitor such as reserpine or verapamil to validate the specificity of the procedure [48]. Notably, the ABCB1 and ABCG2 transporters are correlated with multidrug chemoresistance by exporting a panel of chemotherapeutic agents, such as paclitaxel, cisplatin, and doxorubicin out of the cell [57,58,59,60]. It should be noted that even though SP is highly enriched for cells sharing common CSC-related features, such as self-renewal, multipotency, and tumorigenicity, the two populations are not always entirely identical [61,62].

The sphere formation assay is a relatively simple in vitro method for dissociating and enriching CSCs based on their anchorage-independent growth and self-renewal abilities [63,64]. It is suitable for isolating both healthy as well as undifferentiated cancer cells. It has been demonstrated that stem cells have the potential to form three-dimensional, multicellular spheres, named tumorspheres, when grown in non-adherent, serum-free conditions. Under the microscope, they appear as rounded spheres of various sizes (approximately 50–250 μM) in which the cells are tightly held together. Distinguishing tumor spheres is one of the most efficient and quick methods for isolating CSCs. A great number of studies report the enrichment of cells possessing enhanced chemoresistance, tumor formation, and self-renewal abilities [65,66,67,68,69].

Finally, one of the most commonly used approaches for the identification and isolation of CSCs is the Aldefluor assay [70]. This technique is a fluorescent reagent system, useful for discriminating CSCs based on their aldehyde dehydrogenase enzymatic (ALDH) activity and is related to the metabolism of BODIPY aminoacetaldehyde (BAAA). Specifically, the Aldefluor assay quantifies the ALDH activity by measuring the conversion of BODIPY aminoacetaldehyde to the negatively charged fluorescence compound BODIPY aminoacetate (BAAA-) [71,72]. Subsequently, BAAA- is retained by the ALDH-expressing cells that can then be easily detected based on their high fluorescence profile. An ALDH inhibitor, diethylaminobenzaldehyde (DEAB), reduces fluorescence and is used as a negative control, ensuring specificity [73]. The Aldefluor assay has been vastly applied for efficient CSC enrichment over the past few years, either alone or, most commonly, in combination with other methods, prominently with cell-surface markers. It should be mentioned that while Aldefluor was initially introduced as a method for identifying cells with high ALDH1A1 activity, a great number of later studies demonstrated that several other ALDH isoforms, such as ALDH1A2, ALDH1A3, ALDH1B1, ALDH2, ALDH3A1, ALDH3A2, ALDH3B1, and ALDH5A1 are also involved in this assay. Consequently, Aldefluor is not isoform-specific, and it is impossible to distinguish which specific enzyme is responsible for the monitored ALDH activity [44,74,75,76,77].

### 2.2. Methods for Characterizing CSCs

**Figure 5:**
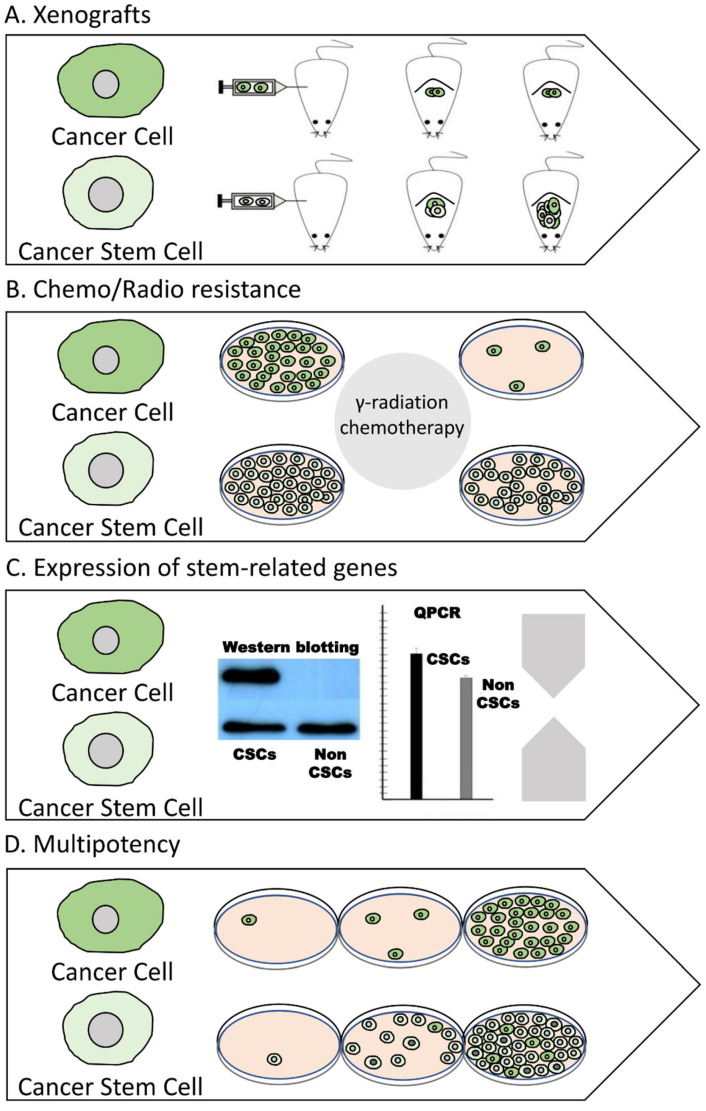
Techniques for evaluating the CSC-related properties of cancer cells. Several methods can be applied to characterize the CSC phenotype of a certain sample of cancer cells. A holistic approach should apply more than one method. (**A**) Assessing the ability of cancer cells to induce heterogeneous tumors in xenograft models. (**B**) Monitoring the endurance against chemotherapeutic agents and/or radiation. (**C**) Examining the regulation of CSC markers. (**D**) Evaluating their multipotency.

Apart from CSC identification, various methods are currently available for evaluating and characterizing the CSC-related phenotype of a certain sample of cancer cells. The most commonly used methods include the xenotransplantation of cancer cells in immune-deficient animals, examination of radio and chemoresistance, evaluation of the expression, activity, and sub-cellular localization of stem-related markers and finally, determination of the multipotency (Figure 5) **[78]**.

A great number of studies support that the xenotransplantation of cancer cells to animal models (xenograft models), typically mice that are immune-compromised (non-obese diabetic/severe combined immunodeficient mice, NOD/SCID mice), is the golden standard for examining the ability of a certain population of cancer cells to initiate tumor growth [79]. There are a number of xenograft models; however, the most prevailing in CSC research are the patient-derived xenografts (PDX) and the cell line-derived xenograft (CDX) models. In these models, mice are engrafted with either patient or cell line-derived cells, respectively [80,81,82,83,84]. It has been shown that CSCs, even when injected at low numbers, have the capacity to efficiently generate tumors that are heterologous and which exhibit similar histological properties as the tumor of origin. These results are repeated with serial tumor transplantations [85,86,87,88].

Radio- and chemoresistance are key features of CSCs and are related to metastasis and disease recurrence [47]. The increased endurance of CSCs against radiation and chemotherapy does not come as a surprise, taking into account the following: their low proliferative or even quiescent state, the altered reactive oxygen species (ROS)-related metabolism, over-expression of drug export pumps, such as members of the ATP-binding cassette (ABC) (e.g., ABCB1 and ABCG2), upregulation of the signaling pathways participating in DNA damage repair (e.g., base-excision repair, homologous/non-homologous end joining), deregulation of apoptosis, and the ability to adopt a mesenchymal phenotype through an epithelial-to-mesenchymal transition (EMT) [89,90,91,92,93,94,95]. A plethora of studies have analyzed the viability and cellular response of CSCs after treatment with a panel of chemotherapeutic agents and/or γ-irradiation both in vitro and in vivo [96,97,98,99]. For instance, patients with a higher frequency of CSCs, as illustrated by higher rates of CSC markers (e.g., ALDH activity), exhibited a poor response to therapy [100,101,102]. Similar results were also obtained in experiments with established/patient-derived cells and patient-derived xenograft models [98,103,104,105,106]. Consequently, in certain studies, chemotherapy enriched the CSC population, while inhibition of the ATP-binding cassette transporter, ALDH and NOTCH reversed the resistance phenotype [99,103,104,106].

Similar to cell-surface proteins, additional markers expressed inside the cell can be utilized to distinguish CSCs from non-CSCs. Some of these proteins, including OCT4, MYC, KLF4, and SOX2 (Yamanaka factors), are universal, considering that they are typical of both cancer and normal stem cells and are considered essential for stem-related phenotypes [107]. The regulation of other markers, though, for instance, NANOG, BMI-1, SNAIL, ALDHs, LGR5, CXCR4, REX-1, Musashi-1, LETM1, and C-met, is considered to be dependent on both the cancer type and the specific phenotype of the CSCs that we want to analyze (e.g., drug-resistant, tumorigenic, metastatic) [35,108,109,110,111,112,113,114,115,116,117]. Studying the expression of specific genes both at the transcriptional and translation level, as well as the activity and localization of these proteins, is useful in assessing the enrichment of CSC properties [118,119,120,121,122,123].

Finally, the ability of asymmetric division is a key property of CSCs. Several approaches can be employed to examine cancer cell multipotency [124,125]. Xenografts are an example of such an experimental approach, considering that inducing a heterogenous tumor in immunodeficient mice entails the existence of cells that possess not only tumorigenicity and self-renewal abilities but also a transdifferentiation potential. Beyond transplantation, another method that can be used is the multicolor lineage-tracing assay. This involves genetic labeling, e.g., through a lentiviral vector, of distinct cellular populations with different fluorescent markers. Subsequently, these cells can either be grown in culture or be used in xenograft transplantations. Either way, their single-cell lineages can be monitored to identify whether they can generate differentiated cellular descendants through in vitro or in vivo asymmetric divisions [85,126,127,128].

## 3. Aldehyde Dehydrogenase 1B1

### 3.1. Aldehyde Dehydrogenases

**Figure 6:**
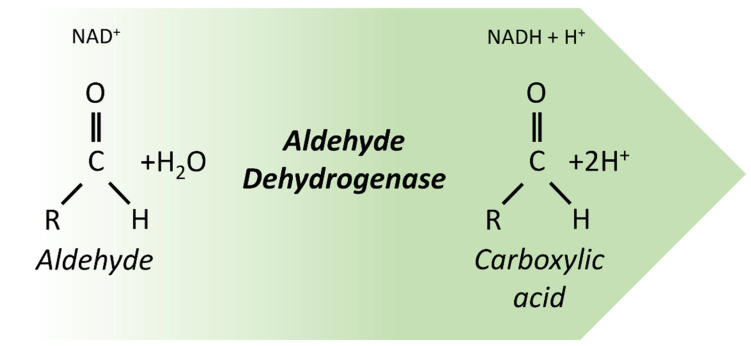
Aldehyde dehydrogenases catalyze the oxidation of an aldehyde to its corresponding carboxylic acid.

Aldehyde dehydrogenases (ALDHs) are a superfamily of nicotinamide adenine dinucleotide (phosphate) (NAD(P)+)-dependent enzymes that catalyze the oxidation of endogenous (lipids, amino acids, and vitamins) and exogenous (ethanol and drugs) aldehydes to their carboxylic acids (Figure 6) [129]. They are multifunctional enzymes with distinct chromosomal locations, which can be found in primates, rodents, birds, fish, and zebrafish [130]. In the human genome, there are 19 ALDH putatively genes, classified into 11 families and 4 subfamilies. Each ALDH isoform exhibits different cellular localizations (cytoplasm, nucleus, mitochondria, endoplasmic reticulum), tissue distributions, substrate specificity, and expression patterns [131]. Moreover, these enzymes have a vital metabolic role in the anti-oxidative defense system through the metabolism of aldehydes. Several ALDH isoforms are associated with RA, betaine, and γ-aminobutyric acid (GABA) production (Figure 6) [129,132].

ALDHs are multifaceted proteins, and apart from their role as metabolic enzymes, they are involved in a plethora of biological processes, such as differentiation, embryogenesis, and DNA damage response [133,134,135]. In addition, ALDHs have been identified as markers of CSC [72,135,136,137,138]. The Aldefluor assay is one of the most commonly used techniques for enriching the CSC population, based on the finding that cells which exhibit high ALDH enzymatic activity (ALDHhigh cells) have enhanced tumorigenicity in various malignancies, including breast [139,140,141,142], liver [143,144], colorectal [145,146,147,148], lung [149,150], prostate [151,152], pancreatic [153,154], ovarian [155], esophageal [156,157], nasopharyngeal [158], gastric [159,160], bone [103,161,162], neuroblastoma [163], skin [164], and blood [165,166] cancer. ALDH activity is characterized by CSC-related characteristics, such as chemo- and radio-resistance [146,167,168,169], hypoxia [170], EMT [146,160], cell proliferation, and invasion [146,147,167]. Aldehyde dehydrogenase 1A1 was one of the first isoforms identified as a CSC biomarker. Since then, research has mainly focused on ALDH1A1 for its ability to metabolize retinaldehyde, its association with normal hematopoietic stem cells, and the idea that Aldefluor specifically isolates cells with high ALDH1A1 activity. However, over the past few years, cumulative evidence highlights the involvement of additional isoforms in Aldefluor activity and the CSC phenotype, in general. Consequently, an accurate experimental approach needs to be able to distinguish the involvement of specific ALDHs, by utilizing, for instance, isoform-specific antibodies and/or real-time PCR analysis. This has led to the identification of a panel of ALDH isoforms that are specifically characterized as CSC biomarkers in different types of cancer. The ALDH1B1 isoform has not been extensively studied, however novel data support its association with cancer development and the CSC phenotype [44,74,75,76,77].

### 3.2. Historical Overview of ALDH1B1 Discovery and Research

The gene of aldehyde dehydrogenase, 1B1, was initially identified in 1991 when Hsu et al. (1991) screened a cDNA library with a synthetic probe of 29 nucleotides based on a conserved amino acid sequence that is found both in ALDH1 and ALDH2 [171]. The authors found that the coding region of the ALDHX gene (later named ALDH5 and, finally, ALDH1B1) is located at the 9.p13.1 chromosomal region and not interrupted by introns [171,172]. The gene encodes a protein of 517 amino acids that exhibits 72% similarity to ALDH2 and 64% similarity to ALDH1A1 [173]. Its high similarity with ALDH2 proved important for gaining information on the origin, the features, and the function of the protein, considering that ALDH2 had been extensively studied and, thus, is better characterized [174,175]. This was utilized by Jackson et al. (2013), who comparatively studied ALDH1B1 and ALDH2 in different vertebrates and created a phylogenetic diagram describing the relationship between these genes [173]. This diagram showed that ALDH1B1 is present only in mammals and amphibians, unlike ALDH2, which is also present in birds and zebrafish. Along these lines, the genomic analysis of human and mouse ALDH1B1 and ALDH2 genes revealed certain similarities, which encouraged researchers to suggest that the ALDH1B1 gene originates from ALDH2 through the retroviral transposition of ALDH2 cDNA into an ancestral chromosome. This evolution process has been described for many vertebrate genes [176,177]. Additionally, the alignment of the amino acid sequences of ALDH1B1 and ALDH2 demonstrated that the essential residues for the function of ALDH2 are also conserved in the sequence of ALDH1B1 [173]. Consequently, the active site of ALDH1B1 is suggested to comprise Cys319 (nucleophile), Glu285 (proton acceptor), and Asn186 (transition state stabilizer), and the NAD-binding residues to be in the area between residues 262 and 267. Furthermore, a mitochondrial localization signal peptide comprising 17 amino acids at the N-terminal of the protein has also been identified [173]. These data corroborate the findings of a previous report by Lutfullah et al. (2011) on the tertiary structure of human ALDH1B1 using the software MODELLER. Based on the structure of ALDH2, there is a high similarity with ALDH1B1 at the residues that are involved in the formation of homotetramers (quaternary structure) [178].

**Figure 7:**
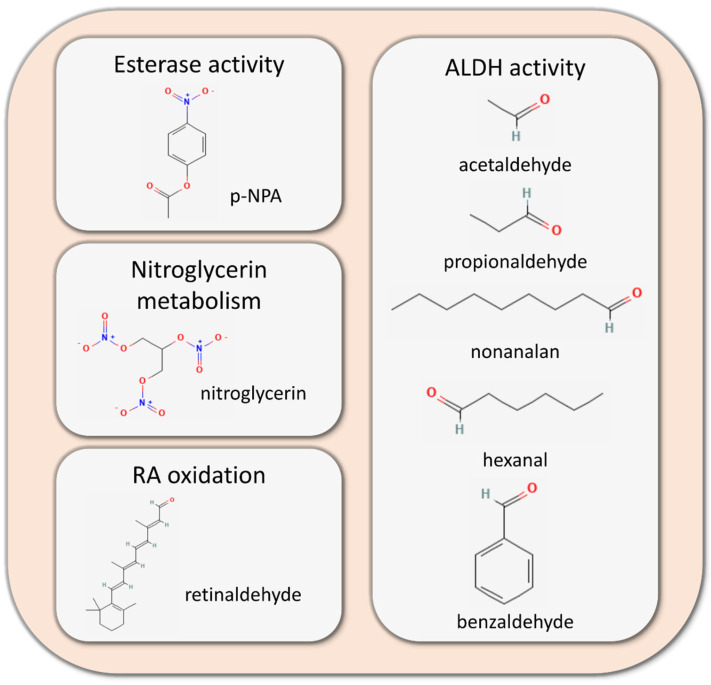
Enzymatic activities of aldehyde dehydrogenase 1B1 (ALDH1B1) (Chemical structures are from PubChem (https://pubchem.ncbi.nlm.nih.gov/) accessed on 5 December 2022) [184,185,186,187,188,189,190,191].

The first in vitro study on the function of human ALDH1B1 was performed by Stewart et al. (1995) and indicated that ALDH1B1 is a NAD-dependent enzyme, in contrast to other ALDH isoforms, which require NADP as a co-factor, and that it exhibits specificity for short-chain aldehydes, such as acetaldehyde and propionaldehyde (Km: 30 μM). The same study also demonstrated that the highest enzymatic activity of ALDH1B1 was observed at mitochondrial pellets, demonstrating for the first time its mitochondrial localization, verifying the potential utility of the mitochondrial localization signal peptide, previously reported by Jackson et al. [179]. Furthermore, Stagos et al. (2010) heterologously expressed human ALDH1B1 in insect cells (Sf9) and purified it through affinity chromatography [180]. The purified protein was used to assess the kinetic properties and specificity of ALDH1B1 against different aldehydes, confirming the specificity of ALDH1B1 for NAD+ (Km = 3.6 μM) as a co-factor and esterase activity of ALDH1B1 in addition to its ALDH function. ALDH1B1 exhibited high specificity for medium-chain aldehydes (hexanal and nonalan with Km values below 1 μΜ), short-chain aldehydes (acetaldehyde with Km = 55 μM and propionaldehyde with Km = 14 μΜ) and aromatic aldehydes (benzaldehyde with Km = 50 μΜ). The esterase activity was identified using p-nitrophenyl acetate (p-NPA) as the substrate, and the Km of the reaction (KmALDH1B1 = 288 μΜ) was estimated to be much higher compared to the respective Km of ALDH2 (ΚmALDH2 = 1895 μM), as reported previously by Sheikh et al. (1997) [181]. In the same perspective, Jackson et al. (2015) applied a similar methodology to Stagos et al. (2010) and determined all-trans retinaldehyde as a substrate of ALDH1B1 with Km = 24.9 μΜ, implying the involvement of ALDH1B1 in the retinoic acid signaling pathway—a finding that was occasionally overlooked in studies focused on RA-related ALDHs (Figure 7) [182]. Finally, recent crystallographic data by Feng et al. (2022) revealed the completed tertiary and quaternary structures of ALDH1B1 [183].

The association of ALDH1B1 with the stem cell phenotype was initially shown by Stagos et al. (2010), using immunohistochemistry to study the distribution of ALDH1B1 in human tissues. ALDH1B1 was mainly expressed in the small intestine, liver, and pancreas and at lower levels in the lung and colon, where its expression appeared to be associated with stem cells [180]. Along these lines, Chen et al. (2011) reported high expression rates of ALDH1B1 in samples of colon adenocarcinoma [192]. Similarly, Singh et al. (2015) revealed that the inhibition of ALDH1B1 attenuated the ability of SW 480 colon adenocarcinoma cells to form spheres in vitro and induce xenograft tumors in vivo while deregulating the Notch, Wnt/β-catenin and PI3K/Akt pathways [145]. ALDH1B1 has also been identified by Ioannou et al. (2013) as a putative marker of progenitor and stem cells in the pancreas, and inhibition of ALDH1B1 activity enhanced the differentiation and led to the formation of smaller embryonic explants [193]. The importance of ALDH1B1 in pancreatic stem cells has been validated by Anastasiou et al. (2016) by constructing mouse aldh1b1 knockout cell lines and demonstrating that the absence of ALDH1B1 led to an acceleration of the differentiation processes, dysregulation of beta cell-related factors, disruption of the function of beta cells and, ultimately, the induction of glucose intolerance [194].

**Figure 8:**
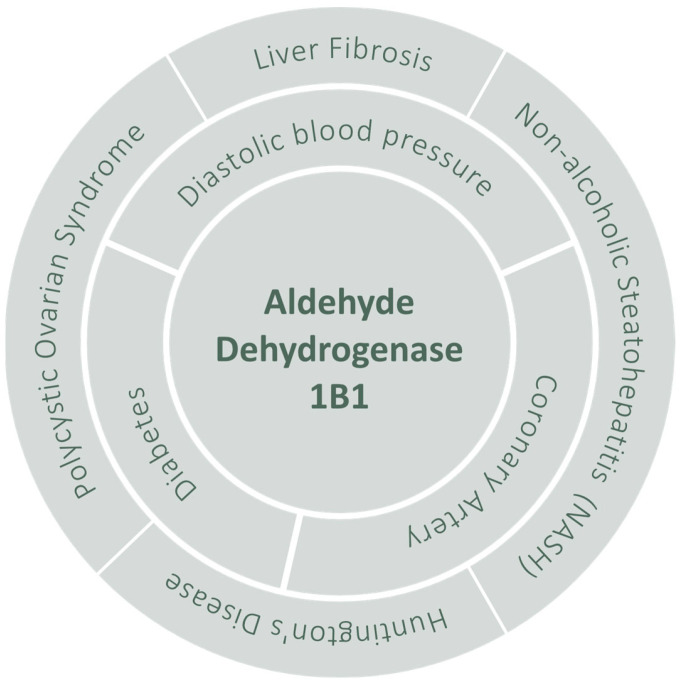
ALDH1B1-associated non-cancer pathologies [195,196,197,198,199,200,201].

There is plenty of valuable information on the properties and biological role(s) of ALDH1B1. Its great importance is highlighted by the multiple pathological conditions, both cancer (Table 1) and non-cancer related (Figure 8), with which ALDH1B1 has been associated over the years. In the following section, we will discuss ALDH1B1′s role in the development of cancer and the CSC phenotype.

## 4. Current Knowledge on the Association of ALDH1B1 with Cancer Progression and CSC Phenotype

### 4.1. Colorectal Cancer

A substantial number of studies are available on the association of ALDH1B1 and colorectal cancer, mainly investigating tumor expression and, in certain cases, in comparison to the respective non-cancerous tissues [145,146,192,204,206,207]. For instance, Chen et al. (2011) suggested that ALDH1B1 can serve as a putative colorectal cancer biomarker on the basis of its high expression levels in human colon cancer tissues [192]. In accordance with these findings, Matsumoto et al. (2017) demonstrated high ALDH1B1 and ALDH2 transcriptional and translational levels in human CRC cell lines and tissue samples [206]. Specifically, six CRC cell lines (BE, Caco-2, COLO320DM, HCT116, HT29, and SW480), as well as patient-derived tissues, were utilized to evaluate the expression levels of eight different ALDH isoforms. Both ALDH1B1 and ALDH2 were expressed in all the cell lines tested, suggesting they play a crucial role in colon cancer [206]. In concordance, Wang et al. (2021) showed that ALDH1B1 expression is significantly higher in colorectal adenomas and adenocarcinomas compared to normal and cancer-adjacent tissues [204]. Golla et al. (2020) took a step further and explored whether ALDH1B1 was essential for colon tumorigenesis by utilizing an inducible mouse model in which the adenomatous polyposis coli gene (APC) is inactivated after exposure to tamoxifen. Generating double knockdowns of ALDH1B1 and APC showed that mice with the absence of 1B1 still formed adenoma colorectal tumors; however, the volume was significantly lower compared to the mice with physiological levels of ALDH1B1. Furthermore, the inhibition of ALDH1B1 expression led to the downregulation of both p53 and b-catenin in these models [205].

These findings are consistent with the utilization of ALDH1B1 as a biomarker, and several studies also support its association with stem-like characteristics, also suggesting its potential utilization as a CSC marker in CRC. Singh and colleagues (2015) demonstrated the implication of ALDH1B1 in different CSC-related cellular signaling pathways, including Wnt/β-catenin, Notch, and PI3K/Akt in CRC [145], and ALDH1B1 shRNA in the colon adenocarcinoma SW480 cell line, led to the downregulation of various proteins, such as LEF1, C-Myc, JAG1, c-Notch1, Akt, PI3K, FABP5, and MMP2 [145]. Langan et al. (2012) showed that high ALDH1B1 expression is significantly associated with poor and/or moderate differentiation and metastasis in patient-derived CRC tumors [207]. We (Tsochantaridis et al. 2021) have shown that ALDH1B1 expression is associated with increased migratory potential, altered cell-cycle regulation and increased chemoresistance (against doxorubicin and 5-fluorouracil (5-FU)), accompanied by ZEB1-related EMT induction as well as lower p21 protein levels in HT-29 cells [146]. Additionally, we (Tsochantaridis et al. 2022) reported that ALDH1B1 expression in HT-29 cells led to resistance against the genotoxic and apoptotic effects of etoposide along with higher total and phosphorylated (Ser15) p53 levels. In the same study, it was demonstrated that ALDH1B1 expression was associated with the upregulation of various DNA damage-related genes [134]. The positive effect of ALDH1B1 on DNA damage repair was also illustrated by Spearman’s rank correlation coefficient analysis, performed with public data from 531 samples of colorectal adenocarcinoma patients [134]. This finding is of importance, considering that the CSCs exhibit highly elaborative and effective DNA damage response machinery [216]. Finally, Baek et al. (2021) reported that AMBRA, a negative regulator of several CSC-related genes, negatively regulates ALDH1B1 through K27- and K33-associated ubiquitination [203].

Given the well-established significance of ALDH1B1 in colon cancer, studies have now started to focus on how to accomplish its inhibition in order to enhance the efficiency of current therapeutic protocols. Feng et al. (2022) identified a group of bicyclic imidazolium that inhibited the activity of ALDH1B1 inside cells. Significantly, both the knockdown of the ALDH1B1 gene through CRISPR, as well as the inhibition of its enzymatic activity resulted in attenuated colon spheroid and xenograft tumor growth, accompanied by the downregulation of colorectal CSC markers, such as the *KRT15* and *DCLK1* [183]. Similarly, Lin et al. (2022) reported that shikonin downregulates ALDH1B1 in a colitis-associated mouse colorectal cancer model [217].

### 4.2. Pancreatic Cancer

Studies by Singh et al. (2016) have demonstrated a high variance of ALDH1B1 expression in a spectrum of 16 different pancreatic cancer cell lines and that ALDH1B1 expression is higher in tissues with more invasive characteristics [153]. Additionally, the knockdown of ALDH1B1 expression through siRNA resulted in significantly lower proliferation rates in vitro and the attenuation of tumorigenicity in xenograft experiments [153]. Centroacinar cells are quiescent, self-renewal progenitors capable of generating various pancreatic lineages and are considered tumor-initiating cells in pancreatic cancer [209]. Such centroacinar cells have been isolated based on their ALDH1B1 expression in adult mice, and the expression of ALDH1B1 was both sufficient and necessary for the maintenance of stem-like properties. ALDH1B1 expression was also positively correlated with the expression of KRAS, an oncogene implicated in pancreatic carcinogenesis, and the expression of ALDH1B1 was necessary for tumor development in a Kras-induced pancreatic mouse model [209].

### 4.3. Other Types of Cancer

ALDH1B1 has been associated with survival in several different types of cancer, leading to contradictory results. For example, Wang et al. (2018) investigated osteosarcoma patient-derived samples and demonstrated a poor prognosis for patients with high ALDH1B1 expression levels [162]. Along these lines, Leung et al. (2017) analyzed the survival data from TCGA and found that ALDH1B1 may be implicated with cancer development in ER-positive breast cancer female patients [215]. Additionally, Zhu et al. (2022) reported that high expression levels of ALDH1B1 are associated with poor overall and progression-free survival rates for patients with nasopharyngeal carcinoma [158]. On the contrary, upregulation of ALDH1B1 at the transcriptional level was significantly associated with higher overall survival in gastric cancer [211], while Yang et al. (2017) demonstrated that high expression levels of ALDH1B1 were correlated with a favorable prognosis in the case of hepatocellular carcinoma [210].

ALDH1B1 also appeared to be implicated with chemoresistance. He et al. (2015) used both A549 and cisplatin-resistant A549/DDP cell lines to assess the expression levels of different ALDH subtypes. Notably, the ALDH1B1 mRNA and protein expression levels were significantly elevated in A549/DDP compared to the A549 indicating the correlation of ALDH1B1 with cisplatin chemoresistance in these cell lines [214]. In accordance, Wang et al. (2018) reported that knocking down ALDH1B1 by siRNA in osteosarcoma cell lines (U2OS and SAOS) resulted in increased chemosensitivity against doxorubicin [162], and Zhu et al. (2022) demonstrated that high ALDH1B1 expression levels in nasopharyngeal carcinoma may be associated with increased patient’s age and increased chemoresistance [158].

Only a few studies have evaluated ALDH1B1 in relation to other CSC-related properties. Yan et al. (2014) investigated a panel of ALDH isoforms for identifying potential prostate cancer biomarkers and found that ALDH1B1 did not exhibit any significant association with the DU145 cancer stem-like cells (spheres) in comparison with the other ALDH isoforms tested [151]. Hartomo et al. (2013) analyzed the expression levels of the ALDHs genes through real-time PCR in the tumorspheres of three different neuroblastoma cell lines (NBTT2D, NBTT1, and NBTT3). Their results indicated that ALDH1B1 was downregulated in neuroblastoma NBTT3-derived spheres as compared to differentiated NBTT3 cells; however, there was no significant difference in the expression levels between the differentiated- versus stem-like NBTT2D and NBTT1 cells [218]. Similarly, Wang et al. (2018) performed siRNA-mediated knockdowns of ALDH1B1 in osteosarcoma cells (U2OS and SAOS) and reported that, in the absence of ALDH1B1, both cell lines exhibited slower proliferation, invasion, and migration potentials. Additionally, ALDH1B1 silencing induced apoptosis through the upregulation of cleaved caspase 3 and cleaved caspase 9, and led to G1-phase cell-cycle arrest compared to the control group. The inhibition of ALDH1B1 was also related to the downregulation of various CSC markers, such as CD44, NANOG, OCT4, SOX2, and NOTCH1 in U2OS cells. Interestingly, ALDH1B1 shRNA attenuated the ability of the U2OS cells to form tumors in xenograft experiments, illustrating its importance in the tumor-initiating process [162]. Another study of the same group (Wang et al. 2020) demonstrated that ALDH1B1 is negatively regulated by microRNA-761; miR-761 appeared to suppress tumor formation in the xenograft models and regulate cell adhesion, EMT, and TGF-β by targeting ALDH1B1 in osteosarcoma [219]. Finally, Chen et al. (2022) identified that the translation initiation factor, EIF4E, promotes ferroptosis by downregulating ALDH1B1 and, thus, leading to 4-HNE accumulation. This finding indicates that the inhibition of ALDH1B1 could support the effectiveness of anticancer ferroptosis inducers [220].

## 5. Conclusions

Research on CSCs has come a long way since the hierarchical model of cancer development was initially introduced. A wide range of methodologies and techniques are currently available for efficiently enriching CSCs and characterizing their properties. However, we are far from fully understanding their origins, properties, and behaviors, and thus, crucial parts are missing from obtaining a glimpse of the whole picture of how cancer actually “works”. Identifying biomarkers and, most significantly, understanding how and why these markers are specifically expressed by the CSCs, will provide us with valuable information on the molecular mechanisms underlying the biology of cancer. It will provide us with novel targets for extending and upgrading the effectiveness of our anticancer arsenal. It is well established that ALDHs have a key role in the CSC phenotype, and even though the vast majority of studies were, for a long time, focused on the ALDH1A1 isoforms, we now know that additional ALDH isoforms contribute to the CSC phenotype. However, it is currently unknown why distinct ALDH isoforms have been “selected” in different cancer types. ALDH1B1 is considered a valid marker of colon carcinogenesis and cancer stem-like phenotype, but it also appears to be associated with normal colon stem cells. Similarly, in the pancreas, though supported by limited experimental data, ALDH1B1 appears to be involved in both differentiation- and carcinogenesis-related processes. Although ALDH1B1 has not been extensively studied in other types of cancer, the experimental evidence available so far supports its emergence as a promising candidate marker of CSCs. Indeed, ALDH1B1 antioxidant activity, its association with the metabolism of RA, its potential to upregulate the DNA damage response cascade, and its involvement with cancer and stem-related pathways, such as Wnt/β-Catenin, Notch, and PI3K/Akt, can provide several advantages to cancer cells. Along these lines, novel findings also highlight ALDH1B1 as a promising therapeutic target for ameliorating the therapeutic outcome of anticancer treatments. Further studies are now required to clarify its exact role in stem cell properties, cancer initiation, and progression.

## Figures and Tables

**Table 1:** Studies on the association of ALDH1B1 with cancer.

Type of Cancer	Techniques Used	Samples Used	Ref.
Colorectal Cancer	Computational analysis	Colon and rectum adenocarcinoma (TCGA samples)	[202]
	Western blot/Flow cytometry/PCR/Comet assay	Cell line/TCGA samples	[134]
	shRNA/Immunoblot/qPCR	Cell line	[203]
	Immunohistochemistry/Computational analysis	Patient and tissue samples	[204]
	Macroscopic evaluation/Immunohistochemistry	Xenografts	[205]
	shRNA/Western blot	Cell lines	[145]
	qPCR/Western blot/Flow Cytometry	Cell line	[146]
	qPCR/Western blot/Aldefluor assay	Cell lines/Tissue	[206]
	Immunohistochemistry	Tisue	[192]
	Colorectal Tissue Micro Array/Immunohistochemistry	Patients	[207]
	p53 lentiviral shRNA	Colon cancer stem cells	[208]
Pancreatic Cancer	FACS/Immunofluorescence	In vivo	[209]
	shRNA/Immunohistochemistry/RT-PCR	Cell lines/Tissue	[153]
Osteosarcoma	siRNA/Flow cytometry/Western blot	Osteosarcoma patients/Cell lines/In vivo	[162]
Nasopharyngeal Carcinoma (NPC)	Immunohistochemistry/Bioinformatic analysis	Patients	[158]
Hepatocellular Carcinoma	Kaplan–Meier survival analysis	Patients	[210]
Gastric Cancer	Kaplan–Meier survival analysis	Patients	[211]
	qPCR	MKN-45, SGC-7901 and MKN-45, SGC-7901 spheres	[160]
Prostate cancer	SNPs	Prostate cancer patients	[212]
Esophageal Squamous Cell Carcinoma (ESCC)	Computational analysis	Patients	[213]
Lung Adenocarcinoma/Lung Squamous Cell Carcinoma	Computational analysis	Lung adenocarcinoma/Lung Squamous Cell Carcinoma (TCGA samples)	[202]
	Flow cytometry/Western blot/qPCR	Cell line	[214]
Breast Cancer	Computational analysis	Breast cancer (TCGA samples)	[202]
	RNA-seq/survival data	Breast cancer patients(TCGA)	[215]
Glioblastoma	Computational analysis	Glioblastoma (TCGA samples)	[202]

## Data Availability

Not applicable.
